# Rifampin-induced fluvastatin toxicity: a case report and review of literature

**DOI:** 10.1093/omcr/omag144

**Published:** 2026-08-03

**Authors:** Ahmed Farghali, Hesham Mahmoud, Muhammad Salem

**Affiliations:** Department of Clinical Pharmacy, Hamad General Hospital, P.O. box 3050, Doha, Qatar; Department of Clinical Pharmacy, Hamad General Hospital, P.O. box 3050, Doha, Qatar; Department of Clinical Pharmacy, Hamad General Hospital, P.O. box 3050, Doha, Qatar

**Keywords:** Fluvastatin, rifampin, CYP2C9, OATP1B1, drug interaction, rhabdomyolysis

## Abstract

Fluvastatin is extensively metabolized by CYP2C9, which is induced by rifampin over several weeks, leading to a concentration decline. However, since rifampin is a potent inhibitor of the primary first-pass hepatic statin uptake transporter, the organic anion transporting polypeptide 1B1 (OATP1B1), simultaneous administration has been shown to significantly increase statin exposure. We report a previously fluvastatin-tolerant 63-year-old woman with nonalcoholic steatohepatitis who developed statin-induced rhabdomyolysis four weeks after the initiation and co-administration of rifampin for tuberculosis. After both medications were stopped, the patient showed clinical and biochemical improvement. While this report is the first to describe a clinical outcome of this interaction, multiple pharmacokinetic studies have demonstrated opposing, time-dependent rifampin effects on statin concentrations. Safe management of this challenging interaction includes statin dose reduction and/or separate administration upon rifampin initiation until the anticipated onset of CYP induction, followed by careful and gradual dose titration with monitoring of both effectiveness and toxicity.

## Introduction

Fluvastatin, a low- to moderate-intensity lipid-lowering 3-hydroxy-3-methylglutaryl coenzyme A (HMG-CoA) reductase inhibitor, is approved by the U.S. Food and Drug Administration for the treatment of familial hypercholesterolemia, homozygous in adults and heterozygous in patients aged ≥10 years, and for reducing the risk of atherosclerotic cardiovascular disease as both primary and secondary prevention [1]. Additionally, statins are recommended to reduce cardiovascular morbidity and mortality risk in patients with nonalcoholic fatty liver disease (NAFLD) [2].

Although almost completely absorbed (>90%), indicating minimal intestinal metabolism and limited efflux, fluvastatin oral bioavailability ranges from 19–29% due to extensive first-pass hepatic extraction mediated primarily by the rate-limiting hepatic influx transporter, organic anion transporting polypeptide 1B1 (OATP1B1). Subsequently, it is predominantly metabolized (>80%) by cytochrome P450 2C9 (CYP2C9) [3]. Decreased-function polymorphisms in solute carrier organic anion transporter 1B1 (*SLCO1B1*), which encodes OATP1B1, and *CYP2C9* are associated with increased fluvastatin systemic exposure and reduction of its maximum tolerated dose [4].

Rifampin is an essential antimicrobial used in the management of several serious infections, including prosthetic valve infective endocarditis caused by coagulase-negative staphylococci and tuberculosis [5]. As a potent inducer of multiple CYPs, uridine 5′-diphospho-glucuronosyltransferases (UGTs), and ATP-binding cassette (ABC) efflux transporters [5], rifampin is a major perpetrator of numerous drug–drug interactions, mainly reducing systemic exposure of substrates, including statins, to extremely low levels [1, 6–11]. However, it is also a strong competitive inhibitor of the predominant hepatic statin influx transporter OATP1B1, which may result in increased statin concentrations [11–21]. These opposing effects, described in multiple pharmacokinetic studies [1, 6–21], make rifampin interaction with statins difficult to manage, as they may result in either markedly reduced or elevated statin levels, leading to either attenuation of lipid-lowering effect or increased risk of muscle toxicity, respectively.

Specifically, rifampin induces *CYP2C9* transcription by activating its key *de novo* synthesis regulatory nuclear receptor, pregnane X receptor, thereby increasing its mRNA expression rate up to six-fold [5]. However, the time course required for CYP2C9 induction ranges from two to more than 4 weeks for maximal effect, which can delay the need for fluvastatin dose escalation [5, 22]. Conversely, there is evidence that single-dose and simultaneous rifampin co-administration with fluvastatin can significantly increase fluvastatin exposure [14, 16].

This report aims to describe a case of rifampin-induced fluvastatin toxicity following the addition of anti-tuberculous therapy in a patient who had been receiving chronic fluvastatin treatment.

## Case report

A 63-year-old woman with a 10-year history of type 2 diabetes mellitus, hypertension, and cirrhosis due to nonalcoholic steatohepatitis (NASH) was admitted to Hamad General Hospital in Doha, Qatar, on September 1, 2023, following referral from a private hospital due to abnormal findings on imaging, including pleural effusion, pulmonary consolidation, and enlarged mediastinal lymph nodes. Her chronic home medications included amlodipine, perindopril, sitagliptin, dapagliflozin, ferrous sulfate, rabeprazole, and fluvastatin 80 mg daily. Prior to admission, she had experienced a five-day history of intermittent fever, for which she received unspecified intravenous antibiotics with no improvement, accompanied by generalized body aches, fatigue, and malaise.

Computed tomography (CT) scan was suspicious for either malignancy or tuberculosis. Since the patient was unable to produce sputum for acid-fast bacilli (AFB) testing, she underwent pleural fluid aspiration for analysis and culture. The results indicated exudative fluid with elevated protein and lactate dehydrogenase levels, yet the AFB test was negative. A pan-CT scan, performed on September 4, revealed right-sided moderate to severe pleural effusion with underlying consolidation, but no significant mediastinal lymphadenopathy. On September 10, video-assisted thoracoscopy with pleural biopsy confirmed tuberculosis. The patient was initiated on standard anti-tuberculous therapy—rifampin, isoniazid, pyrazinamide, and ethambutol—and was discharged with video directly observed therapy (vDOT).

On October 10, she presented to the emergency department with a three-day history of persistent generalized weakness, bilateral lower limb weakness, and palpitations. Blood tests revealed markedly elevated liver enzymes, with alanine aminotransferase (ALT) at 132 U/L and aspartate aminotransferase (AST) at 575 U/L. Since ultrasound Doppler and CT pulmonary angiogram ruled out sepsis, dehydration, and venous thromboembolism, her symptoms were attributed to the anti-tuberculous medications, which were held, and she was discharged.

However, on October 18, she was readmitted with progressive lower limb weakness over the preceding week, progressing to complete inability to walk. Her liver enzymes were further elevated to ALT 285 U/L and AST 732 U/L. Additionally, creatine kinase (CK) and myoglobin levels exceeded 14 000 U/L and 10 000 ng/mL, respectively. She was initiated on intravenous hydration, and the infectious diseases team recommended switching to second-line anti-tuberculous therapy: moxifloxacin, ethambutol, and amikacin. Magnetic resonance imaging (MRI) of the head and spine was performed to evaluate for possible tuberculous myelitis or drug-induced myopathy, which suggested myositis. However, electromyography (EMG) findings did not support this diagnosis. Given the atypical features, including rapid onset of symptoms, involvement of distal muscles, and sensory impairments, the rheumatology team suspected that the symptoms might be drug-induced. When asked specifically by the clinical pharmacist, the patient’s son confirmed that she had been administering rifampin and fluvastatin simultaneously, within the same hour of vDOT, raising the possibility of rifampin-mediated inhibition of fluvastatin hepatic uptake. As a result, the clinical pharmacist recommended the immediate cessation of fluvastatin. An MRI of lower limbs revealed multiple areas of intramuscular edema and intermuscular fluid collections in both thighs. The combined clinical, laboratory, and imaging findings suggested that rifampin-induced fluvastatin-associated rhabdomyolysis was the most likely explanation, which was further supported by both the gradual clinical and biochemical improvement following fluvastatin cessation. On November 16, CK and myoglobin levels decreased from greater than 14 000 U/l and 10 000 ng/ml to 276 U/l and 149 ng/ml, respectively.

## Discussion

To our knowledge, this is the first real-world report describing a clinical outcome of the statin–rifampin interaction. Nonetheless, multiple pharmacokinetic studies in healthy-volunteers have examined the combination. Whereas atorvastatin, fluvastatin, pravastatin, and rosuvastatin have been evaluated following both simultaneous and separate rifampin administration, pitavastatin and simvastatin have only been investigated following simultaneous or separate rifampin administration, respectively [1, 6–21]. Studies employing multi-day separate rifampin courses have reported various degrees of reduction in statin AUC, attributed to rifampin’s potent induction of metabolizing enzymes and efflux ABC transporters, including CYP3A4, CYP2C9, UGTs, P-glycoprotein (P-gp), multi-drug resistant protein 2 (MRP2), and breast cancer resistant protein (BCRP) [1, 6–11]. In contrast, studies involving single-dose or multi-day simultaneous rifampin and statin administration have demonstrated significant increases in statin exposure [11–21]. Since all statins undergo extensive OATP1B1-mediated first-pass hepatic extraction followed by intrahepatic metabolism and/or clearance through ABC transporters [3, 22], rifampin’s timely competitive inhibition of the influx transporter may compromise hepatic uptake, leading to significantly increased statin concentrations [11–21].

Specifically, three studies evaluated fluvastatin–rifampin interaction: one reported that six days of rifampin use reduced fluvastatin single-dose AUC by 53% [1], whereas the other two demonstrated that simultaneous single-dose administration of both agents was associated with up to 2.6-fold increase in fluvastatin AUC [14, 16]. Consistently, most other studies of simultaneous statin–rifampin administration described comparable magnitude of increases in statin AUC and *C_max_* [14–21]. Notably, unlike other statins, single-dose simultaneous rifampin has not been shown to paradoxically shorten fluvastatin half-life, likely due to comparable reduction in apparent volume of distribution at steady state (*Vss/F*) and apparent clearance (*CL/F*); however, this does not negate the significant interaction-induced multifold increase in statin exposure, either for fluvastatin or for other statins [16]. Table 1 summarizes all previous pharmacokinetic studies of statin–rifampin interaction.

**Table 1:** Previous pharmacokinetic studies of statin–rifampin interaction.

Statin	Metabolic enzyme(s) and/or ABC transporter(s)	Study author, year	Healthy volunteers (n)	Rifampin dose^a^ ^,^ ^b^ (mg) X duration	Statin dose (mg) X duration	Timing of administration	AUC ratio (+/− rifampin)
**Atorvastatin**	CYP3A4, BCRP, P-gp	He YJ (2009) [12]	6^c^	600 × 1	40 × 1	Simultaneous	833%
		Lau YY (2007) [13]	11	600 × 1	40 × 1	Simultaneous	782%
		Takehara I (2018) [14]	8	600 × 1	1 × 1	Simultaneous	610%
		Tatosian DA (2021) [15]	6^d^	600 × 1	0.1 × 1	Simultaneous	608%
		Pfizer (2019) (Label) [11]	Not specified	600 × 7	40 × 1	Simultaneous	112%
		Backman JT (2005) [6]	10	600 × 5	40 × 1	Separate	20%
**Fluvastatin**	CYP2C9	Xiang Y (2021) [16]	10	600 × 1	20 × 1	Simultaneous	260%
		Takehara I (2018) [14]	8	600 × 1	2 × 1	Simultaneous	250%
		Sandoz (2023) (Label) [1]	Not specified	600 × 6	20 × 1	Separate	53%
**Lovastatin**	CYP3A4	Not studied	-	-	-	-	-
**Pitavastatin**	UGT1A3, UGT2B7	Chen Y (2013) [17]	12	600 × 1	4 × 1	Simultaneous	640%
		Prueksaritanont T (2014) [18]	8	600 × 1	1 × 1	Simultaneous	570%
		Tatosian DA (2021) [15]	6^d^	600 × 1	0.01 × 1	Simultaneous	367%
		Takehara I (2018) [14]	8	600 × 1	0.2 × 1	Simultaneous	280%
		Kowa (2024) (Label) [19]	Not specified	600 × 5	4 × 5	Simultaneous	129%
**Pravastatin**	MRP2	Deng S (2009) [20]	12	600 × 1	20 × 1	Simultaneous	227%
		Kyrklund C (2004) [8]	10	600 × 5	40 × 1	Separate	69%
		Lutz JD (2018) [7]	20	600 × 12	20 × 3	Separate	42%
**Rosuvastatin**	BCRP	Prueksaritanont T (2014) [18]	8	600 × 1	5 × 1	Simultaneous	437%
		Tatosian DA (2021) [15]	6^d^	600 × 1	0.05 × 1	Simultaneous	356%
		Wu HF (2017) [21]	15^c^	600 × 1	20 × 1	Simultaneous	329%
		Takehara I (2018) [14]	8	600 × 1	0.5 × 1	Simultaneous	240%
		Zhang W (2008) [9]	18	450 × 6	20 × 1	Separate	96%
		Lutz JD (2018) [7]	20	600 × 14	10 × 5	Separate	37%
**Simvastatin**	CYP3A4	Kyrklund C (2000) [10]	10	600 × 5	40 × 1	Separate	13%

AUC, area under the concentration–time curve; BCRP, breast cancer resistance protein; CYP, cytochrome P450; MRP2, multi-drug resistant protein 2; P-gp, P glycoprotein; UGT, Uridine diphosphate-glucuronosyltransferase.

^a^Highest rifampin dose data.

^b^Oral or IV, reported oral rifampin data in studies using both.

^c^Wild-type *SLCO1B1* data.

^d^Healthy-volunteers data.

Among all, atorvastatin stands out with the highest single-dose rifampin-associated AUC increase, up to 8-fold, following same-time co-administration [12–15]. Interestingly, only a 12% increase in AUC was observed when a single atorvastatin dose was simultaneously administered with the final dose of a 7-day rifampin course [11]. Similarly, whereas single-dose rifampin simultaneous administration with pitavastatin was associated with a 2.8–6.4-fold AUC increase [14, 15, 17, 18], a 5-day simultaneous pitavastatin–rifampin administration resulted in only a 29% increase [19]. These markedly attenuated increases are likely ascribed to time-dependent development of pharmacokinetically apparent CYP3A4, UGT, BCRP, and P-gp induction [23], which could have limited atorvastatin and pitavastatin absorption through enhancing pre-portal intestinal metabolism and apical-to-lumen efflux, as well as increasing intra-hepatic metabolism and biliary excretion. Since the separate administration of the same atorvastatin dose following a 5-day rifampin course was associated with an 80% decrease in AUC, the manufacturer recommends simultaneous administration to maintain therapeutic concentrations [11]. However, the consequences of prolonged simultaneous statin–rifampin administration have never been investigated. Extended co-administration may result in either subsequent progressive statin accumulation due to consecutive OATP1B transporter inhibition or concentration decline following the time required for maximal enzyme and/or ABC transporter induction.

In our case, a previously fluvastatin-tolerant patient at the maximum dosing level, symptoms of myopathy, including muscle weakness and severe myalgia, developed 27 days after concomitant rifampin use with simultaneous administration. Since rhabdomyolysis was laboratorically confirmed, a diagnosis of statin-induced muscle toxicity was established, suggesting that rifampin phenoconverted the patient to fluvastatin-intolerant. Whereas the conventionally expected outcome of such a combination is reduced fluvastatin serum concentration due to potent CYP2C9 induction by rifampin [1], toxicity, suggesting accumulation, was instead observed. This can be attributed to rifampin’s inhibition of OATP1B1-mediated hepatic uptake of fluvastatin. However, the net outcome of influx transporter inhibition taking precedence over enzyme induction despite crossing the usual time course to significant enzyme abundance is yet to be mechanistically explained.

**Figure 1:**
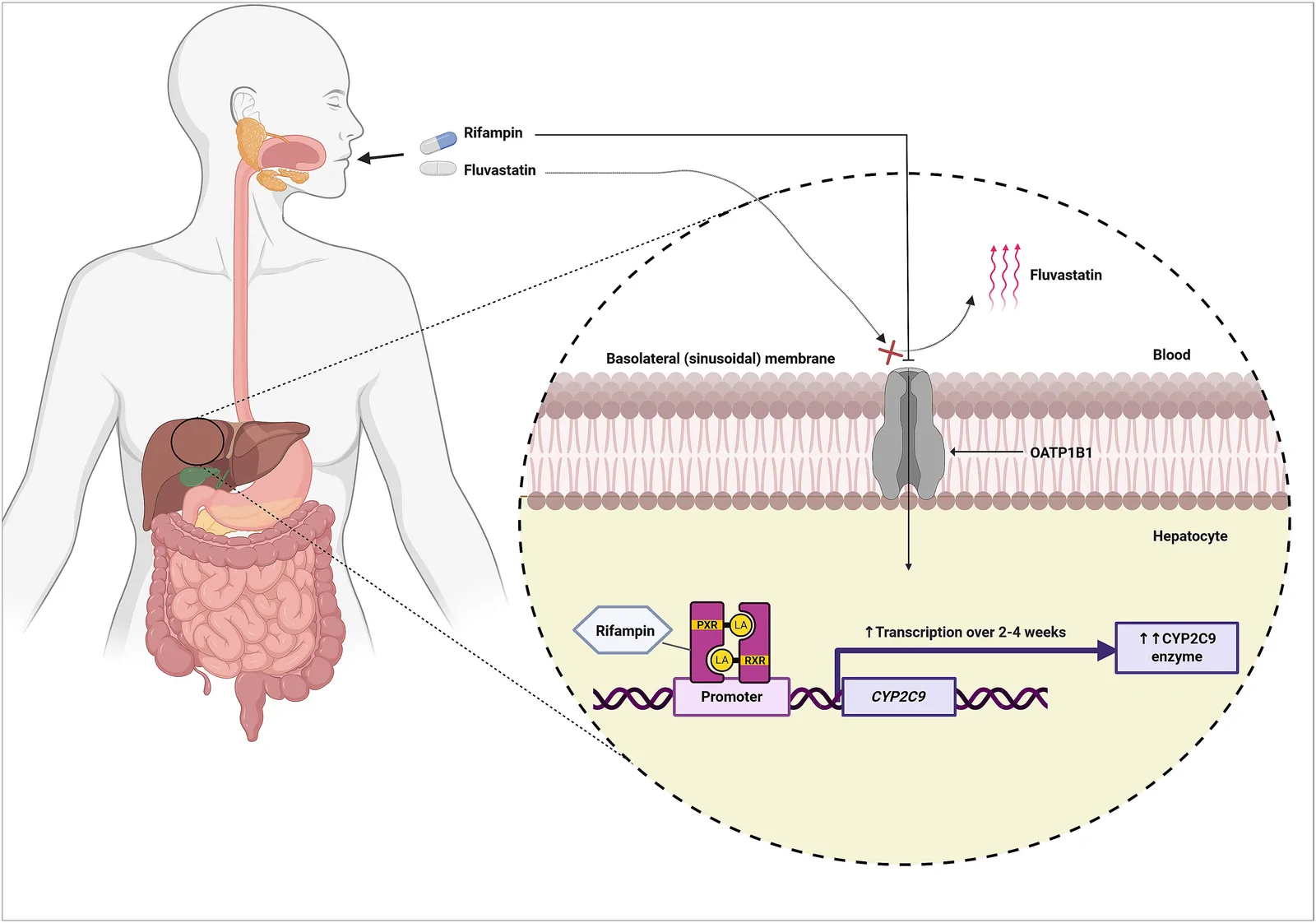
Suggested mechanism of rifampin-induced fluvastatin accumulation. Following simultaneous rifampin and fluvastatin administration, rifampin competitively inhibits first-pass OATP1B1-mediated hepatic uptake of fluvastatin, leading to systemic accumulation. Concurrently, inside the hepatocyte, rifampin activates the pregnane X receptor via ligand binding, forming a heterodimer with retinoid X receptor. This complex binds to the *CYP2C9* promoter, resulting in increased CYP2C9 enzyme expression. However, CYP2C9 induction requires 2–4 weeks to reach maximal enzyme abundance. Created in BioRender. Salem, M. (2025) https://BioRender.com/k7pqbus. CYP, cytochrome P450; LA, ligand-activated; OATP, organic anion transporting polypeptide; PXR, pregnane X receptor; RXR, retinoid X receptor.

One explanation is that rifampin’s OATP1B inhibition, upon simultaneous administration, blocked the way of fluvastatin first-pass hepatic extraction and subsequent intrahepatic metabolism, leading to accumulation. The interaction continued over the initial weeks from co-initiation to toxicity, even while the CYP induction process had already commenced. Fluvastatin, in comparison to other statins, has unique pharmacokinetics, being extensively dependent on CYP2C9. Since CYP2C9 is markedly less expressed in the intestine than CYP3A4, 15% versus 80%, respectively [24], this may result in less pronounced post-induction intestinal first-pass metabolism. Although BCRP, induced by rifampin, may moderately contribute to fluvastatin transport [25], evidence is limited for a major role in overall fluvastatin clinical exposure [4, 26]. Additionally, CYP2C9 has the longest turnover half-life among CYP enzymes and efflux transporters [5, 23]. Most reported cases of rifampin-CYP2C9 induction have required more than 2 weeks to become clinically apparent, and up to 4–5 weeks for maximal induction [5]. The delayed CYP2C9 induction, along with timely OATP inhibition, may explain our case’s presentation with rifampin-induced fluvastatin myopathy within one month of co-administration. Suggested mechanism of rifampin-induced fluvastatin accumulation is illustrated in Fig. 1.

Another possible explanation might involve *CYP2C9* and/or *SLCO1B1* decreased-function genetic polymorphisms, which have been associated with elevated risk of fluvastatin-associated musculoskeletal symptoms [4]. While the patient had shown persistent tolerance to the maximum fluvastatin dose at baseline, carrying one or more of these variants may increase the risk without necessarily causing overt toxicity. If such polymorphisms are present, this may reveal a pre-interaction high-risk of musculoskeletal toxicity, which could have been exacerbated by rifampin.

Notably, He *et al*. demonstrated that single-dose rifampin increased co-administered atorvastatin AUC in subjects with decreased function homo- and heterozygous *SLCO1B1* genotypes by ratios of 330% and 468%, respectively, versus 833% in wild-type [12]. Nevertheless, the absolute AUC levels with rifampin were comparable across genotypes, likely due to the lower baseline atorvastatin AUC values in individuals with wild-type compared to variant carriers [12]. In the case of fluvastatin, *CYP2C9* variants may, additionally, lead to excessive exposure while using simultaneous rifampin, particularly at the initiation phase of the latter, when influx transporter inhibition precedes full metabolizing enzyme induction.

It remains uncertain whether a similar clinical outcome would result from the simultaneous co-administration of other statins with rifampin. However, safer strategies during rifampin initiation include employing separate administration, utilizing half the previously tolerated dose or half the maximum genotype-dependent Clinical Pharmacogenetics Implementation Consortium (CPIC) guidelines recommended dose [4], or withholding the statin during the first 1–2 weeks of the combination, depending on the anticipated time course to significant induction of metabolizing enzymes or efflux transporters. Thereafter, gradual statin dose escalation and/or cautious simultaneous co-administration of reduced statin dose with rifampin may be considered to achieve adequate lipid-lowering response, with close monitoring for signs of myopathy.

In conclusion, statin–rifampin interaction management remains exceptionally challenging due to the dual mechanism and sensitive time course. Our report demonstrated that, during the rifampin initiation phase, simultaneous administration with fluvastatin may lead to statin accumulation and severe muscle toxicity. Temporarily withholding fluvastatin or utilizing separate administration followed by gradual dose escalation after crossing the anticipated onset of CYP2C9 induction may offer a safer approach. Prospective, extended studies are warranted to determine the optimal statin dosing and timing of co-administration with rifampin to maintain safe and effective lipid-lowering therapy.
